# Contraceptive dynamics among women with disabilities of reproductive age in Ethiopia: systematic review

**DOI:** 10.1186/s13643-024-02456-w

**Published:** 2024-01-26

**Authors:** Abebe Alemu Anshebo, Yilma Markos, Sujit Behera, Natarajan Gopalan

**Affiliations:** 1 Department of Epidemiology and Public Health, School of Life Sciences, Central University of Tamil Nadu, Thiruvarur, India; 2 Deparment of Midwifery, College of Medicine and Health Sciences, Wachemo University, Hosanna, Ethiopia; 3 Department of Public Health, College of Medicine and Health Sciences, Wachemo University, Hosanna, Ethiopia

**Keywords:** Contraceptives, Family planning, Reproductive age, Women with disabilities, Ethiopia

## Abstract

**Background:**

In low-income countries, women with disabilities have limited access to essential sexual and reproductive health services and are disadvantaged socioeconomically. Even though some studies have been conducted previously, there are scanty findings on contraceptive use and associated factors among women with disabilities. Thus, this systematic review aimed to assess contraceptive use and associated factors among women with disabilities of reproductive age in Ethiopia.

**Methods:**

The Preferred Reporting Item for Systematic Review and Meta-Analyses [PRISMA] guidance is used to conduct this systematic review. Data were searched from electronic databases: PubMed/Medline, Scopus, Google Scholar, and other relevant sources. Studies screening was done using Rayyan software. The findings were narratively synthesized using a socio-ecological framework for health promotion.

**Result:**

Ten cross-sectional studies and 4436 women with disabilities of reproductive age were included in this review. According to this review, women with disabilities are less likely to use contraceptives, with a prevalence of 21.7% in Gondor City and 44.4% in Addis Ababa. The associated factors were identified and themed at individual, interpersonal, community, and institutional levels.

**Conclusion:**

Overall, the review findings revealed that women with disabilities continue to encounter challenges ranging from individual level to disability-unfriendly health facility infrastructure or institutional level. Therefore, health professionals and other relevant stakeholders should draw attention to creating awareness towards contraceptive use at individual and interpersonal levels, ensuring accessible contraceptive services and disability-friendly health facilities.

**Supplementary Information:**

The online version contains supplementary material available at 10.1186/s13643-024-02456-w.

## Background

Disability is defined by the World Health Organization (WHO) as any personal condition that prevents a person from living a normal daily life cycle, and it can be clarified as any one of difficulties or not functioning well, activity limitation, impairment, or participation limitations [1, 2].

According to the World Report on Disability (WHO and World Bank 2011), 15% of the global population experiences at least one type of disability, and as a result, people with disabilities lack adequate health care, have inaccessible information, have low educational performance, stigma, and discrimination, lack active participation in decision-making and high rate of poverty [3, 4].

In Ethiopia, based on World Report on Disability, 15 million people live with disabilities, accounting for 17.6% of the population, and 95% of the population with disabilities is estimated to live in poverty [5, 6]. The cause of disability is multidirectional, and people with disabilities are disadvantaged socioeconomically and marginalized to access essential healthcare services [7]. In low-income countries, 80% of the population with disabilities has limited access to basic sexual and reproductive health services [8].

The United Nations (UN), by the General Assembly, 2019, reaffirmed the commitment to assure universal health coverage (UHC) for all, including accessing equitable sexual and reproductive health (SRH) services [9]. In the Sustainable Development Goals (SDGs) agenda, sexual and reproductive health and reproductive rights are featured and aimed to assure universal health for all [10]. However, people with disabilities were not mentioned and focused on this goal, even though the UN General Assembly 2019 pledges to “leave no one behind” [11]. For Women with disabilities, lack of access to sexual and reproductive services is about violation of the basic right to appropriate services. Furthermore, it could hinder activities to assure health access for all as proposed in SDG 3 [12].

Decision-making around contraceptive use is fundamental for women to ensure sexual and reproductive health. However, women with disabilities have faced numerous barriers to making their own decisions to uptake the contraceptive services [13]. Globally, women with disabilities have substantial unmet needs concerning reproductive and sexual health services [2]. Women with disabilities are marginalized in sexual and reproductive health services [14]. Access to affordable, quality sexual and reproductive services is fundamental to realizing the well-being of women [15]. Contraception is one of the strategies that can enable women and men to decide freely and responsibly the number and spacing of their children and to ensure informed choices [16]. Universal access to contraceptive methods helps women to have planned pregnancies and avoid the adverse health and socioeconomic consequences of unintended pregnancy and abortion [17].

Studies revealed that women with disabilities have low contraceptive use and were associated with poor socioeconomic status, lack of awareness towards sexual and reproductive health, inaccessible health services and disablity-unfriendly health facilities, stigmatization, and healthcare providers’ perceptions [18, 19]. Even though some research has been conducted on contraceptive use and associated factors among women with disabilities of reproductive age [20, 21], there is no systematic review on contraceptive use among women with disabilities in Ethiopia that provides higher-level evidence for local actors, donors, beneficiaries, and policy-makers and to support interventions. The findings of this systematic review can provide insights for health care providers, programmers, researchers, and policy-makers and also provide the best possible and up-to-date scientific evidence for all.

Therefore, this systematic review explored and synthesized the available scientific evidence on contraceptive use and associated factors among women with disabilities of reproductive age in Ethiopia.

## Methods

### Registration

The systematic review is registered with International Prospective Registry of Systematic Review (PROSPERO) (No: CRD42023395088).

### Search strategies

This systematic review has adhered to the Preferred Reporting Item for Systematic Review and Meta-Analyses (PRISMA) guideline and checklist [22]. The electronic databases PubMed/Medline, Scopus, and Google Scholar were searched for relevant studies. Additionally, research institutions’ archives were explored for unpublished articles, and likewise, backward citation searching was used to find relevant studies. Studies that reported contraceptive utilization, unmet needs, and associated factors among women with disabilities of reproductive age were searched. Studies conducted from July 2013 to July 2023 were included in this systematic review to explore and synthesize up-to-date scientific evidence. We used the key terms and Boolean Operators “OR” and “AND” to explore all relevant studies from sources (see Supplementary file 1).

### Eligibility criteria

All studies that met the inclusion criteria were included in this systematic review, and the eligibility criteria were based on the condition, context, and population (COCOPO) principles (Table 1).

**Table 1:** The inclusion and exclusion criteria of the systematic review

**Criteria**	**Inclusion**	**Exclusion**
Population	Women with disabilities of reproductive age	Women without disabilities
Condition	Contraceptive or family planning use, unmet need, and associated factors	
Context	Ethiopia	Other countries
Type of disabilities	Women with physical or visual or hearing disability	Women with developmental disability or dual disabilities
Time	2013-2023	Before 2013
Type of study	All observational studies	Interventional studies

### Study screening

**Fig. 1:**
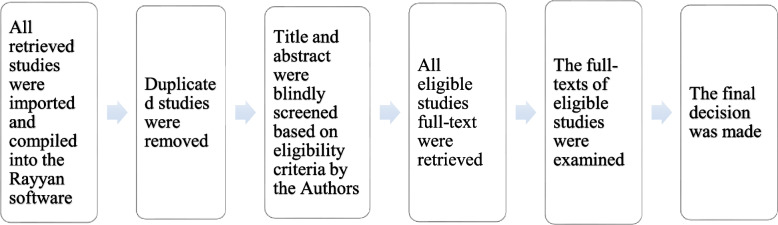
The selection process of eligible studies included in this systematic review

All records retrieved by searching the electronic databases and other sources were merged and imported to Rayyan software and then screened independently by two authors (AA and YM). Discussion among team members helped to achieve consensus on including studies where uncertainty existed regarding their eligibility (Fig. 1).

### Outcome of interest

In this review, the outcome of interest was contraceptive use among women with disabilities of reproductive age in Ethiopia. Contraceptive utilization is defined as who has ever used or is currently using at least one modern contraceptive method. Individual, interpersonal, community, institution, and public policy level factors associated with contraceptive use were secondary outcomes of interest in this systematic review.

### Critical appraisal

The suitability of full-text articles for inclusion was evaluated independently by two authors (AA and YM). The Newcastle–Ottawa Scale adapted tool for cross-sectional studies for systematic review was used to assess the quality of all included articles [23]. The tool evaluates each study’s sample size, sample representativeness, non-respondents, ascertainment of the risk factors, comparability of subjects, assessment of outcome, and statistical tests. Two authors (NG and SB) assessed the quality of the articles. The tool has a scoring system for each article included in the review and classified it into either unsatisfactory quality (0–4, out of 10), satisfactory quality (5-6, out of 10), good quality (7-8, out of 10), or very good quality (9-10, out of 10). All articles included in this review scored at least satisfactory quality (see Supplementary file 2).

### Data extraction and synthesis

The team created a data extraction Excel spreadsheet that consists of the publications’ author name, year of publication, study region, area, design, sample size, prevalence, associated factors, type of disability women living with, and contraceptive method used. To ensure accuracy, two authors (AA and YM) did data extraction independently and checked by a third author (SB). Inconsistencies in extracted data were handled by having a thorough discussion with team members. The data were gathered and synthesized narratively from the data presented in the studies reviewed. There was heterogeneity in outcome measurement and related factors in the studies reviewed, leading to a lack of data suitable for meta-analysis. Thus, this narrative synthesis adhered to synthesis without meta-analysis (SWiM) reporting guidelines [24] and thematically adopted a socio-ecological framework for health promotion [25, 26].

## Result

### Search result

**Fig. 2:**
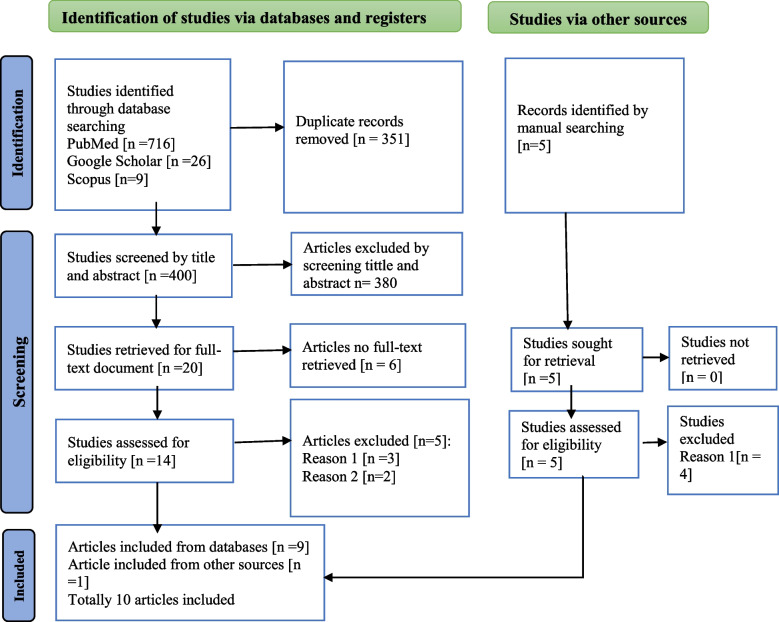
PRISMA flow diagram describes the selection of study for a systematic review on contraceptive use and associated factors among women with disabilities in Ethiopia 2013–2023. Reason 1: studies included young people with disabilities. Reason 2: studies included male and female living with disabilities

In total, 756 studies were retrieved from the search strategies, 751 from the electronic databases, and five from searching other sources. Of these, 351 were identified as duplicate records and removed, and 400 studies were screened by title and abstract based on eligibility criteria, with 380 records being excluded. Following the full-text review of the remaining 20 studies, 10 were assessed as meeting the eligibility criteria and were included in this review (Fig. 2).

### Characteristics of included studies in review

All of the studies in this review were conducted using a cross-sectional study design and included 4436 women with disabilities of reproductive age. Of 10 studies, nine were conducted in the community setting, but one was done at the health facility level [27]. Furthermore, nine studies were published, and one study was not published [28]. The estimation of contraceptive prevalence varies from study to study, with the prevalence of contraceptive use ranging from 21.7% in Gondor City [29] to 44.4% in Addis Ababa [28]. The study that had the most participants was conducted in Addis Ababa (*N* = 727) [28], while the study with the least participants was conducted in Jimma town (*N* = 206) [30]. Forty percent of the studies were conducted in the Amhara region, and 20% in Addis Ababa, Ethiopia (Table 2).

**Table 2:** Description of the studies included in the systematic review, 2013–2023, Ethiopia (*N* = 10)

**Author**	**Region**	**Setting**	**Design**	***N***	**Prevalence**
Tenawu et al. (2023) [18]	Sidama	Community	Cross-sectional	620	27.3%
Mesfin Yesgat et al. (2020) [31]	SNNPR	Community	Cross-sectional	418	33.7%
Beyene et al. (2019) [29]	Amhara	Community	Cross-sectional	280	21.7%
Terefe et al. (2022) [30]	Oromia	Community	Cross-sectional	206	47.1%
Mekonnen et al. (2020) [32]	Amhara	Community	Cross-sectional	412	24.5%
Tessema et al. (2015) [27]	Amhara	Institutional	Cross-sectional	337	24.3%
Yimer and Modiba (2019) [33]	Addis Ababa	Community	Cross-sectional	330	31.1%
Abera et al. (2016) [28]	Addis Ababa	Community	Cross-sectional	727	44.4%
Rade et al. (2023) [34]	Amhara	Community	Cross-sectional	567	28.2%
Kellali T, Hadush G, (2017) [35]	Mekele	Community	Cross-sectional	539	27.2%

*N* = sample size

### Factors associated with contraceptive use

In this review, the factors associated with contraceptive utilization were identified, collated, and grouped into five themes according to the social-ecological model for health promotion [25, 26]; individual, interpersonal, institutional, community, and public policy factors and the direction of effect of each factor was mentioned (Table 3).

**Table 3:** Factors associated with contraceptive use among women with disabilities 2013–2023, Ethiopia

*Theme*	*Factors*	*Overall direction of effect*	*Evidences*
**Individual level**	Age [27–29, 33]	Positive	Age group 26–35 and 36–45 versus 15–25 years: AOR = 5.43 (95% CI: 1.71–17.81) and AOR = 3.6 (95% CI: 1.11–11.32) respectively [29]. Age group 35–48 and 25–34 years versus 15–24 years: AOR = 3.0 (95% CI:1.48–5.98) and AOR = 4.3.7 (95% CI: 1.90–10.04) [33]
	Education [27–29]	Positive	Women education: AOR = 5.0 (95% CI: 1.87–13.40) [29], and partner education AOR = 3.1 (95% CI: 1.04–9.50) [28]
	Marital status (married) [29, 31–33, 35]	Positive	Marital status (married): AOR = 2.13 (95% CI: 1.01–4.52) [29], AOR = 3.9 (93% CI: 2.31–6.63) [31] and AOR = 18.4 (95% CI: 10.38–32.50) [35] respectively
	Employment [28, 31]	Positive	Employment: AOR = 2.2 (95% CI: 1.77–4.15) [31], and unemployment: AOR = 0.8 (95% CI: 0.3–2.05) [28]
	Having knowledge about contraceptives methods [27, 32, 33]	Positive	Having knowledge on contraceptive: AOR = 3.31 (95% CI: 1.37–7.59) [32] and AOR = 2.82 (95% CI: 1.47–5.40) [33]
	Positive attitude towards contraceptives [31, 33]	Positive	Positive attitudes: AOR = 2.3 (95% CI: 1.21–3.87) [31]
	Autonomy to visit health facility [34]	Positive	Women autonomy: AOR = 3.30 (95% CI: 1.45–6.92) [34]
	Being subjected to media [34]	Positive	Subjected to media: AOR = 5.9 (95% CI: 1.45–6.92) [34]
**Interpersonal level**	Perceived positive family attitude [33]	Negative	Family attitude: AOR = 0.48 (95% CI: 0.26–0.88) [33]
	Discussion with partner [28, 34]	Positive	Discussion: AOR = 3.3 (95% CI: 1.08–10.34) [28] and AOR = 9.36 (95% CI: 3.44–17.17) [34]
	Living with sexual partner [34]	Positive	Sexual partner: AOR = 9.2 (95% CI: 2.84–13.60) [34]
	Perceived family economy (rich and medium) [29]	Positive	Family economy level: AOR = 11.6 [95% CI: 2.81–47.31] [29]
Community level	Living with disabilities, [18, 30]	Negative	Living with disabilities: AOR = 0.38 (95% CI: 0.18–0.79) and AOR = 0.06 (95% CI: 0.03–0.12) [18]
Institutional level	Transport accessibility to health facilities [18]	Positive	Transport: AOR = 2.28 (95% CI: 1.53–3.94) [18]
	Presence of nearby health facilities [35]	Positive	Nearby health facilities: AOR = 4.04 (955 CI: 2.05–7.93) [35]
	Customer trust in health facility about confidentiality [35]	Positive	Confidentiality: ARO = 2.26 (95% CI: 1.12–4.57) [35]
	Being visited by health care provider [30]	Positive	Healthcare provider visiting reduces unmet need of contraceptive use (AOR = 0.25, 95% CI: 0.09–0.65) which inversely means enabling women to use contraceptive services [30]
Policy level	All studies did not report public policy-related factors that either enable or hinder contraceptive use among women with disabilities in Ethiopia		

Positive direction = enabling factor. Negative direction = hindering factor

#### Individual level factors

Participants’ age is one of the individual-related factors associated with contraceptive use among study participants. Studies revealed that participants aged 25–48 years were significantly associated with contraceptive use [18, 28, 29, 33]; inversely, an age group less than 25 was negatively associated with contraceptive use among women with disabilities [29, 33]. Out of ten studies, three studies reported that women and partner education educational status were positively associated with contraceptive use [27–29]; similarly, five studies highlighted that being married was a positive predictor for contraceptive utilization [27, 29, 31–33]. In this review, studies reported that employment, having a positive attitude, and knowledge of contraceptive methods were significantly associated with contraceptive use [18, 28, 31, 33].

#### Interpersonal level factors

Studies included in this review reported that family-positive attitudes towards contraceptive service [31], discussion and living with sexual partners [29, 34], and having a perceived medium or high economy [29] were associated positively with contraceptive use among women with disabilities.

#### Community-level factors

The community stigma towards women living with disabilities hinders access to appropriate modern contraceptive utilization [18, 30].

#### Institutional level factors

Studies reported that contraceptive utilization among women with disabilities of reproductive age was significantly associated with health facilities and transport accessibility [18, 35], customers’ trust in service provision at health institutions [35], and healthcare provider visits [30].

#### Public policy-level facts

In this review, all included studies did not report public policy-related factors that either facilitate or hinder contraceptive use among women with disabilities in Ethiopia.

### Contraceptive methods used

Four studies reported that most of the participants used contraceptive injections [Depo-Provera]: 65%, 50%, 20.2%, and 93.2%, respectively [29, 31, 34, 35]; on the other hand, two studies reported that implants were the most commonly used method: 48.5%, 51% [18, 33]. Six studies indicated that the least used contraceptive method among women with disabilities was intrauterine contraceptive devices (IUCD): 3%, 4%, 1%, and 0%, respectively [18, 29, 31, 33].

## Discussion

Contraceptive use has a significant contribution to make in ensuring universal sexual and reproductive health care for all, particularly for women with disabilities. This review narratively synthesized the results reported in the studies about contraceptive use and associated factors among women with disabilities in Ethiopia, and it included the findings from nine published and one unpublished study.

In this review, the reported prevalence of contraceptive use among women with disabilities varies from 21.7% in Gondor City [29] to 44.4% in Addis Ababa [28]. Similarly, other studies revealed a comparable prevalence of contraceptive use at 26.1% in South Africa [36], 32.0% in Kenya [37], and 34.2% in Uganda [38]. Nevertheless, other studies reported a relatively high prevalence of contraceptive use: 59.3% in Turkey [39], 73.0% in India [40], and 70.1% in the United States of America [41].

The evidence suggests that contraceptive utilization among women with disabilities is low. Beyond the bounds, it indicates that there might be a high risk of unwanted pregnancy and abortion. This finding suggests that more dedication from all concerned bodies is needed to ensure sexual and reproductive health services for all women with disabilities, as pledged by the United Nations to “leave no one behind.”

In five studies, maternal age 25–48 years is associated with contraceptive use among women with disabilities [18, 27–29, 33]; on the other hand, the age group less than 25 was less plausibly associated with contraceptive use [33, 42]. Moreover, another study reported that women aged 25–34 years were reported as contributors to contraceptive use in South Africa [36]. The possible explanation might be that women aged 25–48 years have a high demand for contraceptive utilization. This age range is an idea at which most women get married, have frequent sexual practices with sexual partners, and need contraceptives to space and limit the number of children.

Women and husbands’ literacy level was found to be one of the enabling factors for contraceptive use in this review [27, 28, 42]. Likewise, studies reported that women’s and husbands’ education levels significantly contributed to contraceptive uptake among women with disabilities [36]. It might be that the education may help women and their partners to have awareness, knowledge, and a positive attitude towards contraceptive services and to be autonomous in making decisions on sexual and reproductive health matters. Additionally, education can increase women’s self-esteem and enable them to demand contraceptive services. Thus, accessing education for all women is fundamental to ensuring universal sexual and reproductive health coverage.

In this review, studies reported that married women had higher odds of contraceptive utilization compared to their counterparts [31–33, 35]. This finding converges with the study conducted in Nepal [19]. This evidence implies married women might have more sexual practice with their sexual partner and, as a result, usually need contraceptive methods to limit or space the number of children. Hence, being married may increase the demand for and use of contraceptives among women with disabilities.

Unemployment was reported as a barrier to access to contraceptive use among women living with disabilities in this review [28, 31]. This finding is supported by a systematic review of qualitative studies [43]. This result implies that women’s employment is an enabling factor for contraceptive use and helps them to make decisions on sexual and reproductive health services.

In this review, the findings revealed that contraceptive utilization is significantly associated with women’s knowledge and positive attitudes towards contraceptive methods [18, 31–33]. This finding is comparable to the studies reported in a systematic review [37], in Kenya [43] and Turkey [44]. A possible explanation might be that knowing about contraceptive methods enables women to differentiate the pros and cons of the services. Additionally, a positive attitude may trigger women to use the service effectively. Hence, women’s knowledge and positive attitude towards contraceptives can play a significant role in the uptake of contraceptives.

According to this review, being disabled is a significantly associated factor with contraceptive use among women with disabilities of reproductive age [18, 30]. This finding is supported by a study conducted in the USA [45]. The other way round, other studies revealed that disabilities do not have a significant association with modern contraceptive utilization in the Pakistan Demographic Health Survey [46] and in the USA [47]. The possible explanation for the inconsistency of the findings might be due to differences in the study population’s socioeconomic status, time, and study approach.

The results of this review indicate that women’s autonomy in accessing healthcare facilities and exposure to media are found to be enabling factors for contraceptive use [34]. Similarly, other studies have reported the findings in Nepal [19] and South Africa [36]. The possible explanation might be that the media has made a significant contribution to creating awareness towards sexual and reproductive health matters among women with disabilities of reproductive age. Hence, women who have access to media can have information about contraceptive methods, advantages, disadvantages, side effects, and how it works. Moreover, being autonomous enables women to use contraceptives for what they need to do without interference. Therefore, this calls for more efforts to access sexual and reproductive health-related information to women living with disabilities and to ensure women’s empowerment.

In this review, studies reported that perceived family economy level [42] and structural barriers like health facilities design, distance, and transportation access [18, 35] were associated significantly with contraceptive use among women with disabilities. This finding is supported by evidence from a study done in Senegal [20], Uganda [48], Nepal [19] and the USA [49]. A possible explanation is that a low family economy level or financial limitation negatively affects contraceptive service utilization. Additionally, distance from health facilities and lack of transportation to access health facilities make the burden triple-fold for women with disabilities. This evidence clearly showed that all concerned bodies have to draw attention to tackle these barriers and ensure inclusive health services for all.

In this review, disability non-inclusive or unfriendly health facility structure and healthcare providers’ negative attitudes towards women with disabilities were identified as significant predictors of contraceptive use [35]. Studies firmly supported this finding in different parts of the world, like Uganda [21, 48], Senegal [20], and Nepal [19]. These findings highlighted that disability-friendly health facilities and healthcare providers’ good attitudes are essential enabling factors for contraceptive utilization among women with disabilities. Therefore, all health facilities must be structurally inclusive, and healthcare providers must be compassionate to serve all customers without discrimination.

The result of this review indicates that designing need-based behavioral change communication strategies to enhance women’s and community misconceptions and awareness of contraceptive services is needed to overcome the challenges that women with disabilities have been facing [18, 31, 35]. Additionally, other studies have shown that ensuring the availability and accessibility of contraceptive services, compassionate patient-centered care, and disability-friendly health facilities can help to improve contraceptive use among women with disabilities [34, 42].

## Strength and limitation

The strong points of this review are following the PRISMA 2020 guidelines for systematic reviews and using a tool modified for the Newcastle-Ottawa Scale to evaluate the quality of the included studies. In this review, narratively synthesized findings from studies about contraceptive use and associated factors among women with disabilities of reproductive age to provide insight for future research in this area. Contrarily, the data in this review, which was narratively synthesized without meta-analysis, does not reveal any information about the pooled prevalence of contraceptive use among women with disabilities. There might be missed articles because some databases can not be accessed freely.

## Conclusion

According to this review, women with disabilities are less likely to use contraceptives, with a prevalence of 21.7% in Gondor City and 44.4% in Addis Ababa. The associated factors were identified and themed at individual, interpersonal, community, institutional, and public policy levels. Overall, the review findings suggest that women with disabilities continue to encounter challenges ranging from individual attitudes to disability-unfriendly health facility infrastructure. Therefore, all concerned bodies have to pay more attention to creating awareness towards contraceptives at individual and interpersonal levels, ensuring accessibility of contraceptive services and disability-friendly health facilities. Moreover, further studies should be conducted to explore the causal relationship between factors and outcome of interest, public policy-related factors, and to evaluate the programs and policies implemented so far to ensure universal sexual and reproductive health coverage for all.

### Supplementary Information

**Additional file 1:** **Supplementary file 1. **Data searching strategies.

**Additional file 2:** **Supplementary file 2.** Newcastle-Ottawa quality assessment scale for studies included in this review.

## Data Availability

All data pertaining to this review are contained in this document.

## Abbreviations

PROSPERO: International Prospective Registry of Systematic Review
SWiM: Synthesis without meta-analysis
SDGs: Sustainable Development Goals
SRH: Sexual and Reproductive Health
UN: United Nations
UHC: Universal Health Coverage
WHO: World Health Organization
