# Supplementary material for: Contraceptive dynamics among women with disabilities of reproductive age in Ethiopia: systematic review
Source: Syst Rev. 2024 Jan 26;13:42. doi: 10.1186/s13643-024-02456-w (PMC10811808; doi:10.1186/s13643-024-02456-w)
Supplement: Supplementary file 1 — Additional file 1: Supplementary file 1. Data searching strategies. [file 13643_2024_2456_MOESM1_ESM.docx]

**Supplementary file-1: Data searching strategies**

| Database | Search strategy |
| --- | --- |
| PubMed =716 | (((((((((("contracept"[All Fields] OR "contracepted"[All Fields] OR "contracepting"[All Fields] OR "contraception"[MeSH Terms] OR "contraception"[All Fields] OR "contraceptions"[All Fields] OR "contraceptive agents"[Pharmacological Action] OR "contraceptive agents"[Supplementary Concept] OR "contraceptive agents"[All Fields] OR "contraceptive"[All Fields] OR "contraceptive devices"[MeSH Terms] OR ("contraceptive"[All Fields] AND "devices"[All Fields]) OR "contraceptive devices"[All Fields] OR "contraceptive agents"[MeSH Terms] OR ("contraceptive"[All Fields] AND "agents"[All Fields]) OR "contraceptives"[All Fields] OR "contraceptive s"[All Fields] OR "contraceptively"[All Fields]) AND "2013/07/14 00:00":"3000/01/01 05:00"[Date - Publication]) OR (("family planning services"[MeSH Terms] OR ("family"[All Fields] AND "planning"[All Fields] AND "services"[All Fields]) OR "family planning services"[All Fields] OR ("family"[All Fields] AND "planning"[All Fields]) OR "family planning"[All Fields]) AND "2013/07/14 00:00":"3000/01/01 05:00"[Date - Publication]) OR (("contracept"[All Fields] OR "contracepted"[All Fields] OR "contracepting"[All Fields] OR "contraception"[MeSH Terms] OR "contraception"[All Fields] OR "contraceptions"[All Fields] OR "contraceptive agents"[Pharmacological Action] OR "contraceptive agents"[Supplementary Concept] OR "contraceptive agents"[All Fields] OR "contraceptive"[All Fields] OR "contraceptive devices"[MeSH Terms] OR ("contraceptive"[All Fields] AND "devices"[All Fields]) OR "contraceptive devices"[All Fields] OR "contraceptive agents"[MeSH Terms] OR ("contraceptive"[All Fields] AND "agents"[All Fields]) OR "contraceptives"[All Fields] OR "contraceptive s"[All Fields] OR "contraceptively"[All Fields]) AND "2013/07/14 00:00":"3000/01/01 05:00"[Date - Publication])) AND (("statistics and numerical data"[MeSH Subheading] OR ("statistics"[All Fields] AND "numerical"[All Fields] AND "data"[All Fields]) OR "statistics and numerical data"[All Fields] OR "use"[All Fields]) AND "2013/07/14 00:00":"3000/01/01 05:00"[Date - Publication])) OR (("statistics and numerical data"[MeSH Subheading] OR ("statistics"[All Fields] AND "numerical"[All Fields] AND "data"[All Fields]) OR "statistics and numerical data"[All Fields] OR "utilization"[All Fields] OR "utilisation"[All Fields] OR "utilisations"[All Fields] OR "utilise"[All Fields] OR "utilised"[All Fields] OR "utilises"[All Fields] OR "utilising"[All Fields] OR "utilities"[All Fields] OR "utility"[All Fields] OR "utilizations"[All Fields] OR "utilize"[All Fields] OR "utilized"[All Fields] OR "utilizer"[All Fields] OR "utilizers"[All Fields] OR "utilizes"[All Fields] OR "utilizing"[All Fields]) AND "2013/07/14 00:00":"3000/01/01 05:00"[Date - Publication])) AND ("Reproductive-age"[All Fields] AND "2013/07/14 00:00":"3000/01/01 05:00"[Date - Publication]) AND (("womans"[All Fields] OR "women"[MeSH Terms] OR "women"[All Fields] OR "woman"[All Fields] OR "women s"[All Fields] OR "womens"[All Fields]) AND "2013/07/14 00:00":"3000/01/01 05:00"[Date - Publication])) OR (("womans"[All Fields] OR "women"[MeSH Terms] OR "women"[All Fields] OR "woman"[All Fields] OR "women s"[All Fields] OR "womens"[All Fields]) AND "2013/07/14 00:00":"3000/01/01 05:00"[Date - Publication])) AND (("disabilities"[All Fields] OR "disability"[All Fields] OR "disabled persons"[MeSH Terms] OR ("disabled"[All Fields] AND "persons"[All Fields]) OR "disabled persons"[All Fields] OR "disabled"[All Fields] OR "disablement"[All Fields] OR "disablements"[All Fields] OR "disabling"[All Fields] OR "disablity"[All Fields]) AND "2013/07/14 00:00":"3000/01/01 05:00"[Date - Publication])) OR (("disabilities"[All Fields] OR "disability"[All Fields] OR "disabled persons"[MeSH Terms] OR ("disabled"[All Fields] AND "persons"[All Fields]) OR "disabled persons"[All Fields] OR "disabled"[All Fields] OR "disablement"[All Fields] OR "disablements"[All Fields] OR "disabling"[All Fields] OR "disablity"[All Fields]) AND "2013/07/14 00:00":"3000/01/01 05:00"[Date - Publication])) AND (("Ethiopia"[MeSH Terms] OR "Ethiopia"[All Fields] OR "Ethiopia s"[All Fields]) AND "2013/07/14 00:00":"3000/01/01 05:00"[Date - Publication])) AND (y_10[Filter]) |
| Google Scholar  n=154 | Contraceptive Or Family planning And Reproductive-age And "Women with Disabilities" and Ethiopia |
| Scopus = 6 | contraceptive OR family AND planning AND reproductive AND women AND disability AND Ethiopia |
| Other sources like African Journal Online, ProQuest, etc. n=5 | Using key term: Contraceptive OR Family planning And Reproductive And age and women And disabilities And Ethiopia |
