# Supplementary material for: Contraceptive dynamics among women with disabilities of reproductive age in Ethiopia: systematic review
Source: Syst Rev. 2024 Jan 26;13:42. doi: 10.1186/s13643-024-02456-w (PMC10811808; doi:10.1186/s13643-024-02456-w)
Supplement: Supplementary file 2 — Additional file 2: Supplementary file 2. Newcastle-Ottawa quality assessment scale for studies included in this review. [file 13643_2024_2456_MOESM2_ESM.docx]

**Supplementary file_2**: Newcastle-Ottawa quality assessment scale for studies included in this review.

| First author and Year of publication | S1 | S2 | S3 | S4 | C | O1 | O2 | Total score |
| --- | --- | --- | --- | --- | --- | --- | --- | --- |
| Tenaw et al., 2023 | * | * | * | * | ** | * | * | 8/10 |
| Mesfin Yesgat et al., 2020 | * | * | * | * | ** | * | * | 8/10 |
| Beyene et al., 2019 | * | * | * | * | * | * | * | 7/10 |
| Terefe et al., 2022) | * | * | * | * | ** | * | * | 8/10 |
| Mekonnen et al., 2020 | * | * | * | * | ** | * | * | 8/10 |
| Tessema et al., 2015 | * | * | * | * | * | * | - | 6/10 |
| Yimer & Modiba, 2019 | * | * | * | * | ** | * | * | 8/10 |
| Abera, et.al, 2016 | * | * | * | * | ** | * | * | 8/10 |
| Rade et al., 2023 | * | * | * | ** | ** | * | * | 9/10 |
| Kellali T, Hadush G, 2017 | * | * | * | * | * | * | * | 7/10 |

*S1-Repreentativeness of the sample, S2- Sample size; S3- Non-respondents; S4- Ascertainment of the exposure; C1- Comparability; O1-Assessment of the outcome; O2- Statistical test*

**The adapted Newcastle-Ottawa scale quality assessment scale for cross-sectional studies*
